# Sortase A Substrate Specificity in GBS Pilus 2a Cell Wall Anchoring

**DOI:** 10.1371/journal.pone.0025300

**Published:** 2011-10-04

**Authors:** Francesca Necchi, Vincenzo Nardi-Dei, Massimiliano Biagini, Michael Assfalg, Annalisa Nuccitelli, Roberta Cozzi, Nathalie Norais, John L. Telford, C. Daniela Rinaudo, Guido Grandi, Domenico Maione

**Affiliations:** 1 Novartis Vaccines and Diagnostics, Siena, Italy; 2 Department of Biotechnology, University of Verona, Verona, Italy; University of Kansas Medical Center, United States of America

## Abstract

*Streptococcus agalactiae*, also referred to as Group B *Streptococcus* (GBS), is one of the most common causes of life-threatening bacterial infections in infants. In recent years cell surface pili have been identified in several Gram-positive bacteria, including GBS, as important virulence factors and promising vaccine candidates. In GBS, three structurally distinct types of pili have been discovered (pilus 1, 2a and 2b), whose structural subunits are assembled in high-molecular weight polymers by specific class C sortases. In addition, the highly conserved housekeeping sortase A (SrtA), whose main role is to link surface proteins to bacterial cell wall peptidoglycan by a transpeptidation reaction, is also involved in pili cell wall anchoring in many bacteria. Through *in vivo* mutagenesis, we demonstrate that the LPXTG sorting signal of the minor ancillary protein (AP2) is essential for pilus 2a anchoring. We successfully produced a highly purified recombinant SrtA (SrtA_ΔN40_) able to specifically hydrolyze the sorting signal of pilus 2a minor ancillary protein (AP2-2a) and catalyze *in vitro* the transpeptidation reaction between peptidoglycan analogues and the LPXTG motif, using both synthetic fluorescent peptides and recombinant proteins. By contrast, SrtA_ΔN40_ does not catalyze the transpeptidation reaction with substrate-peptides mimicking sorting signals of the other pilus 2a subunits (the backbone protein and the major ancillary protein). Thus, our results add further insight into the proposed model of GBS pilus 2a assembly, in which SrtA is required for pili cell wall covalent attachment, acting exclusively on the minor accessory pilin, representing the terminal subunit located at the base of the pilus.

## Introduction

Many bacterial pathogens persist in their environmental niche and can establish a successful infection by attaching themselves via cell wall anchored proteins, such as adhesins and invasins or using long filamentous appendages, named *pili* or *fimbriae* extending out from the bacterial surface [1], [2]. In recent years, these kinds of appendages were described in several strains of *Corynebacterium*, *Actinomyces*, *Enterococci* and in the principal streptococcal pathogens that cause invasive disease in humans [3], [4], [5], [6], [7], [8], [9]. Although their role in disease has not been studied to the same extent as Gram-negative pathogens, some evidence indicates the involvement of these structures in adhesion and attachment to the host cell, interaction with components of the extracellular matrix (ECM), and biofilm formation [10], [11], [12], [13], [14]. Furthermore, in pathogenic streptococcal species, pili are also reported to be promising vaccine candidates [5], [15], [16]. In *Streptococcus agalactiae* (also known as Group B *Streptococcus* [GBS]), the leading cause of neonatal sepsis and meningitis, three structurally distinct types of pili have been identified, each encoded by a distinct genomic island, named Pilus Island 1 (PI-1), Pilus Island 2a (PI-2a) and Pilus Island 2b (PI-2b) [17]. The overall organization of the three islands is similar. Each island contains genes encoding for three structural proteins harboring a (L/I)PXTG motif, where X is any amino acid, and also for two class C sortase enzymes, which catalyze pilus protein polymerization. Sortase-mediated covalent linkages connecting individual pilin subunits within the pilus structure are a peculiar characteristic of all Gram-positive pili and specific sortase enzymes are also responsible for the covalent linkage of the polymer itself to the cell wall peptidoglycan [3], [18].

In Gram-positive species multiple sortases are grouped into four or five classes based on their primary sequences, membrane topology, genomic localization, and specificity for amino acid sequence motifs [19]. Class C sortases represent the largest and most heterogeneous group of Gram-positive sortases and several copies can be present in a genome [3], [20]. All Gram-positive pathogens express a “housekeeping” sortase A (SrtA), which is responsible for the cell wall anchoring of the majority of surface proteins [11], [21]. However, growing evidence shows that SrtA also plays a role in pilus anchoring to the bacterial cell wall in several species, including GBS [12], [18], [22]. Indeed, a recent genetic analysis revealed that this enzyme is able to mediate the permanent anchoring of GBS pilus 2a to the cell wall, using as an anchor protein the minor ancillary protein [17], which seems to be localized at the base of the pilus as revealed by electron microscopy in pneumococcal pili [23].

*In vitro* studies of *S. aureus* SrtA have begun to define the mechanism of transpeptidation. SrtA substrates contain an N-terminal signal peptide (SP) and a C-terminal cell wall sorting signal (CWSS), consisting of an LPXTG sorting motif, followed by a hydrophobic stretch of amino acids and a short positively charged tail [24], [25]. These proteins, once synthesized and exported are retained within the membrane via their C-terminal hydrophobic domain and their positively charged tail [3]. Catalysis occurs through a ping-pong mechanism, where the two substrates, the LPXTG-containing protein and the peptidoglycan cross-bridge, react alternatively in two consecutive steps. SrtA, with its active cysteinyl group, cleaves the peptide bond between the threonine (T) and the glycine (G) residues of the LPXTG motif. The acyl enzyme intermediate formed between SrtA and the C-terminal threonine of the surface protein is resolved through the nucleophilic attack of amino groups provided by the cross-bridge of a peptidoglycan precursor (lipid II). The resulting lipid II-linked surface protein is then incorporated into the cell wall [3], [26]. In addition to the transpeptidation reaction, sortase also catalyzes a hydrolysis reaction *in vitro* in the absence of a nucleophile [27]. It has also been shown that *S. aureus* SrtA activity is dependent on calcium ions, whose presence stimulates sortase activity eightfold, probably by a mechanism that may facilitate substrate binding [28].

In the present study, mutagenesis and biochemical assays have been used to explore specificity and substrate recognition in GBS sortase A, providing the first biochemical characterization of this enzyme in cell wall anchoring of pilus type 2a. Firstly, through *in vivo* mutagenesis we demonstrate that the LPXTG sorting signal of the minor ancillary protein (AP2-2a) is essential for pilus anchoring. Then, by *in vitro* assays we show the exclusive specificity of SrtA in hydrolyzing only the AP2-LPXTG motif. We demonstrate that a highly purified recombinant SrtA is able to catalyze *in vitro* the transpeptidation reaction between a peptidoglycan analogue and the LPXTG motif of AP2-2a, present in fluorescent peptides and in a recombinant AP2 protein. By contrast, the enzyme is not able to catalyze the transpeptidation reaction with substrate-peptides mimicking sorting signals of the other pilus 2a subunits (the backbone protein and the major ancillary protein). These findings further emphasise the specificity of the SrtA reaction and that the cell wall anchoring of GBS pilus 2a occurs through the LPXTG motif of the minor ancillary protein.

## Results

### The sorting signal of the minor ancillary protein (AP2-2a) is essential for pilus 2a cell wall anchoring

Previous genetic studies in GBS have demonstrated that in *srtA* deletion mutants as well as in knock out strains lacking the minor ancillary protein (AP2-2a), pilus expression on the cell surface was reduced, and pili accumulated in the culture supernatant [17]. This data suggested a key role of the housekeeping SrtA in pilus anchoring to the peptidoglycan using the minor ancillary protein as the anchor subunit [17], [18]. It is also known that SrtA attaches cell surface proteins to the lipid II precursor through the sorting signal via a transpeptidation reaction [3], [19], [21].

**Figure 1:**
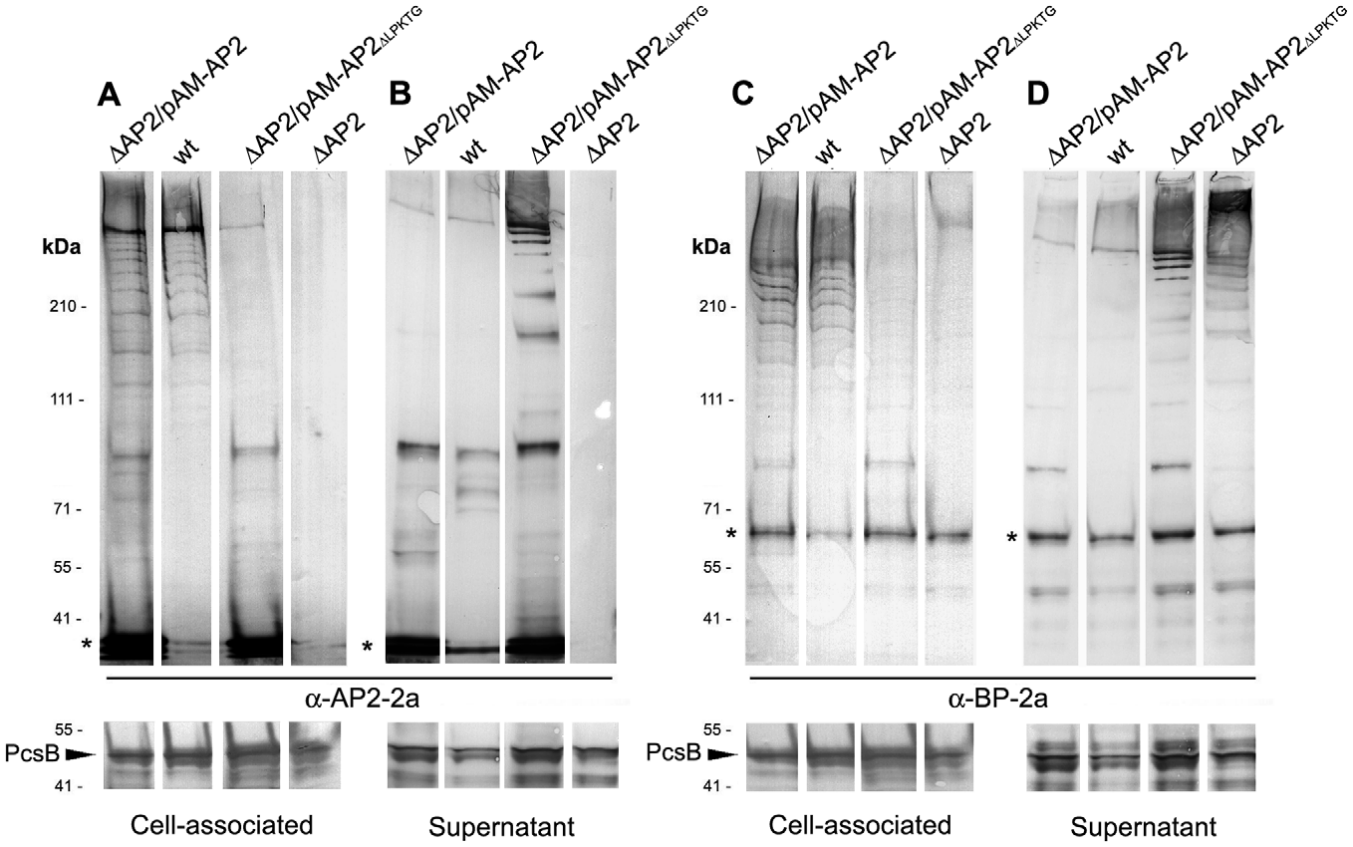
Pilus proteins are mostly released into the supernatant in the AP2-2a_ΔLPKTG_ mutant strain. Proteins were collected from FMC culture supernatants (B and D) or harvested cell pellets (A and C) of GBS strain 515 wild-type (wt), 515 knock-out strain for AP2-2a (ΔAP2) gene and ΔAP2 strains complemented with pAM-AP2_ΔLPKTG_ and with pAM-AP2 wild-type. Protein fractions were analysed by immunoblot stained with antibody specific for AP2-2a (A and B) and for the backbone protein BP-2a (C and D). Asterisks (*) indicate the monomeric form of AP2-2a and BP-2a. The equal quantity loaded in each well is verified by immunoblotting the same gel with a control antiserum that recognizes the protein PcsB of 47 kDa (indicated by a black arrow).

In order to investigate the specific involvement of the C-terminal sorting motif of the ancillary protein AP2 in pilus 2a cell wall anchoring, we generated a complemented GBS strain expressing a mutant form of AP2 protein missing the entire LPKTG motif. By site-specific mutagenesis, we generated a complementation plasmid (pAM_AP2_ΔLPKTG_) to transform the previously described GBS knock-out (KO) mutant strain lacking the AP2-2a gene (515Δ*AP2-2a*) [17]. After complementation, the effects of the deletion on pilus protein polymerization and cell wall anchoring were analysed by Western Blot analysis using equivalent samples representing the bacterial cell wall (Figure 1A and C) and the bacteria-free culture medium (Figure 1B and D), probed with antisera specific for the pilin subunit AP2-2a (Figure 1A and B) and for the backbone protein BP-2a (Figure 1C and D). Deletion of LPKTG peptide in the sorting signal of AP2-2a abolished neither the backbone protein polymerization nor the AP2 incorporation into pili, but higher levels of pilus were released into the culture supernatant (figure 1B and D), similar to the phenotype observed in *srtA* [17] and *AP2-2a* deletion mutants (Figure 1). By contrast, most of the high molecular weight polymers in wild-type 515 strain and in the complemented strain with the plasmid pAM-AP2 (515ΔAP2_pAM-AP2), expressing the wild-type gene, were mainly associated with the cell wall fraction (Figure 1A and C). Thus, the AP2-2a C-terminal sorting signal is dispensable for the incorporation of the minor accessory pilin subunit into pili, but it is otherwise required for the cell wall attachment of the polymeric structure.

### Preparation and characterisation of a Group B *Streptococcus* recombinant SrtA

To characterize in more detail the role and the specificity of GBS SrtA in pilus 2a cell wall anchoring, the catalytic domain of the enzyme was succesfully overexpressed in *E.coli* as a recombinant C-terminal hexahistidine tagged protein. Previous data related to housekeeping SrtA in S. *aureus* and in S. *pyogenes* showed that recombinant forms of both enzymes, lacking the N-terminal 59 and 82 residues respectively, were able to catalyze both the cleavage of LPXTG-containing peptides, and the transpeptidation reaction *in vitro* [27], [29]. Based on the alignment between highly similar SrtA sequences in GBS and S. *pyogenes*, we produced a recombinant form of GBS SrtA from strain 515 encompassing residues from 41 to 247 (SrtA_ΔN40_). The purified enzyme SrtA_ΔN40_ showed >90% purity by SDS-PAGE and analytical gel filtration. Gel filtration/MALLS (Multiple Angles Laser Light Scattering) measurement of SrtA_ΔN40_ revealed that in the analysed peak the protein was mono-disperse with an apparent molecular weight (MW) of 25 kDa, consistent with the theoretical MW of 23.9 kDa of the protein monomer. This data has been further confirmed by means of NMR. The transverse relaxation time (T2), a sensitive indicator of the overall tumbling rate of the molecule, which is directly related to its size, was 22 ms for SrtA_ΔN40_ consistent with the expected MW.

**Figure 2:**
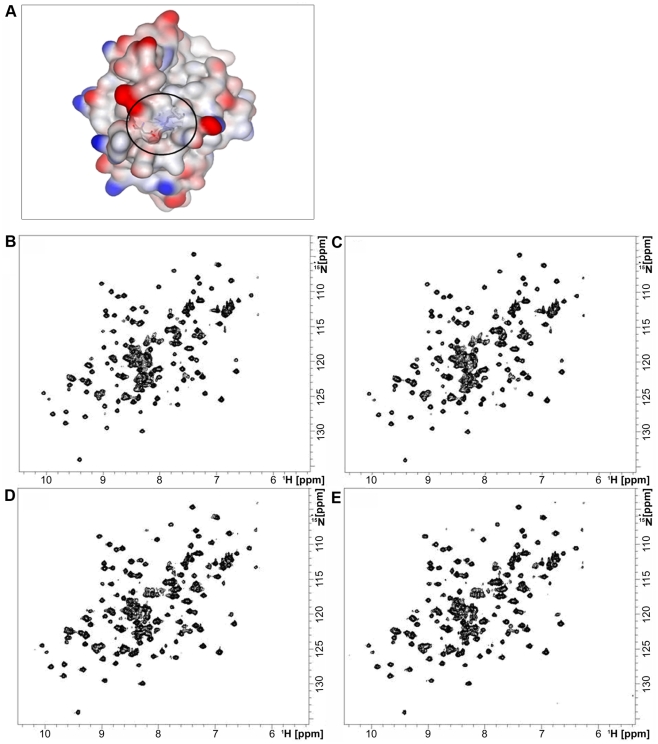
Molecular modeling of SrtA and assessment of metal ion binding by NMR spectroscopy. Surface representation of *S. agalactiae* SrtA showing no similar anionic cleft (black circle) for calcium ion binding, as is the case for *S. aureus* SrtA. In red, negatively charged residues; in blue, positively charged residues (A). ^1^H-^15^N-HSQC spectra recorded on ^15^N-SrtA_ΔN40_ in 30 mM phosphate buffer, 1.5 mM DTT, pH 6.5 (B); 30 mM phosphate buffer, 1.5 mM DTT, 3 mM EDTA, pH 6.5 (C); 50 mM Hepes, pH 6.5 (D); 50 mM Hepes, pH 6.5, CaCl_2_ at a protein∶calcium molar ratio of 1∶10 (E). Spectra were recorded at 600.13 MHz proton Larmor frequency and 298K. Both the addition of EDTA and calcium to the protein did not affect the chemical shift or intensity of the protein signals, indicating that no protein-calcium binding occurs.

*S. aureus* SrtA is the most complete model describing SrtA enzymatic activity. It has been reported that the presence of calcium ions increases the binding of the LPXTG-substrate into the catalytic pocket of the enzyme, as a result of a conformational change of the protein [28]. In particular, in *S. aureus* SrtA_ΔN59_ calcium ion binding is mediated by an anionic cleft near the catalytic site, predominantly formed by two Glu residues (Glu105 and Glu108) and Asp112 on the β3/β4 loop and by Glu171 on the β6/β7 loop, as shown by NMR 1H-15N HSQC (Heteronuclear Single Quantum Coherence) spectra [28]. To explore the structural organization around the catalytic pocket of GBS SrtA, we generated a three-dimensional model of the enzyme, using as a template the crystal structure of *S. pyogenes* SrtA_ΔN81_ (PDB code 3FN5), that shared high sequence identity with GBS SrtA (59% identity and 82.7% similarity). The superimposition between the crystal structure of *S. aureus* SrtA and the GBS SrtA model showed that the calcium binding cleft present in the *S. aureus* enzyme structure appears not to be present in the GBS structural model. In fact, the residues involved in calcium binding were not conserved in the sequence of the GBS enzyme, and in the model, the surface of the pocket was not negatively charged as is the case for *S. aureus* SrtA. Moreover, the whole cleft seemed to be less accessible in the GBS enzyme (Figure 2A).

To experimentally confirm the failure of GBS SrtA_ΔN40_ to bind calcium ions (or other metal cations) we performed NMR spectroscopy measurements. No change in the chemical shift of the amide resonances was observed upon EDTA or calcium ion addition, thus indicating that SrtA_ΔN40_ did not bind to metal ions present in the culture broth or to calcium ions added to the protein solution (Figure 2B–E).

### Group B *Streptococcus* SrtA_ΔN40_ is an active hydrolase and transpeptidase

**Figure 3:**
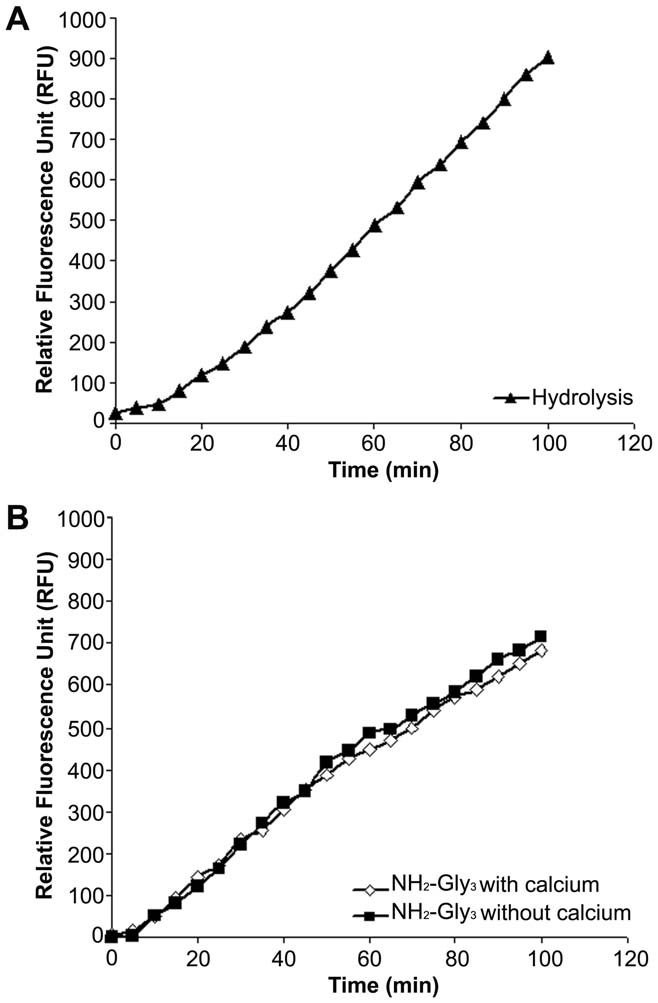
FRET assay for *in vitro* enzymatic activity of SrtA_ΔN40_. (A) Hydrolytic activity of 3 µM SrtA_ΔN40_ with 192 µM fluorogenic peptide Dabcyl-SF**LPKTG**M-Edans in HEPES buffer in which H_2_O is the only nucleophile. (B) SrtA_ΔN40_ (3 µM) catalyzes an *in vitro* reaction in the presence of 75 mM NH_2_-Gly_3_ and 256 µM of fluorogenic peptide Dabcyl-SF**LPKTG**M-Edans. The reaction rate is equivalent with or without 5 mM calcium ions.

In order to evaluate the *in vitro* enzymatic activity of the purified recombinant GBS SrtA_ΔN40_ we performed a Fluorescence Resonance Energy Transfer (FRET) based assay using a synthetic peptide (AP2-2a peptide) mimicking the LPXTG motif of pilus 2a minor ancillary protein (AP2-2a), tagged with the chromophore-quencher pair Dabcyl-Edans (d-SF**LPKTG**M-e) [3], [30]. The peptidase activity of SrtA_ΔN40_ was tested by incubating the enzyme with the selected polypeptide substrate in HEPES buffer in which H_2_O was the only nucleophile. The reaction was monitored over time at 490 nm, and an increase in fluorescence was observed, indicating that the enzyme was able to hydrolyze the AP2-2a fluorogenic peptide (Figure 3A). A fluorescence increase at the same rate was obtained by incubating the tagged polypeptide with the enzyme in the presence of NH_2_-Gly_3_ as a nucleophile, in assay buffers with and without CaCl_2_ (Figure 3B). This shows that the cleavage of the FRET substrate was not dependent either on triglycine or on calcium.

**Figure 4:**
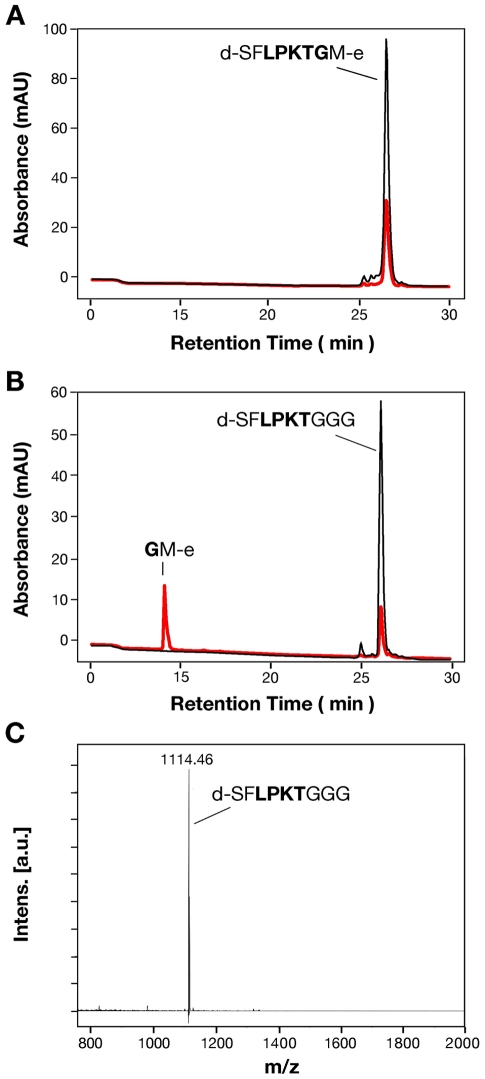
SrtA_ΔN40_ catalyzes *in vitro* transpeptidation reaction. (**A**) RP-HPLC profile of the substrate peptide d-SF**LPKTG**M-e on a C4 column (the LPXTG motif is printed in bold). (**B**) The substrate peptide (20 µM) was incubated overnight with SrtA_ΔN40_ (30 µM) in the presence of 5 mM NH_2_-Gly_3_ at RT. Then the reaction products were separated by RP-HPLC. The eluent was monitored by UV detection at 336 nm (red) and at 472 nm (black). (**C**) MALDI-TOF mass spectra of the reaction product confirmed the predicted molecular weight of the transpeptidation product d-SF**LPKT**GGG.

We further tested the ability of SrtA_ΔN40_ to perform the transpeptidation of the fluorogenic peptide AP2-2a in the presence of NH_2_-Gly_3_. The products obtained from when the reaction was carried out with an excess of enzyme were separated by Reverse Phase-High Pressure Liquid Chromatography (RP-HPLC), and the corresponding fractions were analysed by Matrix-Assisted Laser Desorption/Ionization-Time of Flight Mass Spectrometry (MALDI-TOF MS ) (Figure 4). Two new peaks were identified on RP-HPLC eluting at 14.26 and 26.29 min, respectively, whereas the original peptide peak at 26.51 min disappeared (Figure 4B). The compound that was eluted at 26.29 min generated an *m/z* signal (mass-to-charge ratio) of 1114.46 (Figure 4C), in agreement with the expected MW of the transpeptidation product d-SFLPKTGGG (1113.56 Da). The peak at 14.26 min was not detectable by Mass Spectrometry, but its absorbance at 336 nm and not at 472 nm revealed the presence of the C-terminal Edans, consistent with the fragment GM-e. Neither residual substrate nor hydrolysis products were identified, suggesting that, in the presence of triglycine, SrtA_ΔN40_ completely processed the substrate peptide and the reaction proceeded exclusively towards transpeptidation.

These results demonstrate that recombinant SrtA_ΔN40_ catalyzes the *in vitro* transpeptidation reaction, by cleaving between the threonine and the glycine of the LPXTG motif of AP2-2a. In contrast to *S. aureus* SrtA_ΔN59_ [28], [30], the enzymatic activity is not increased by calcium ions.

### Analysis of SrtA_ΔN40_ substrate specificity with fluorogenic peptides

**Figure 5:**
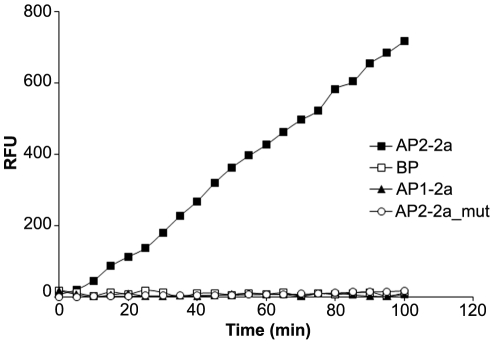
FRET assay with PI-2a peptides for substrate specificity analysis of SrtA_ΔN40_. The reaction solutions contained 256 µM of fluorescent peptide, 3 µM of SrtA_ΔN40_ and 75 mM of triglycine as peptidoglycan analogue. The reactions were performed at 37°C in the assay buffer containing 20 mM HEPES, pH 7.5. We monitored fluorescence emission every 5 minutes and we observed an increase in fluorescence intensity only in the presence of AP2-2a peptide (filled squares), as opposed to BP-2a peptide (open squares) and AP1-2a peptide (filled triangles). In the presence of an additional variation of AP2-2a motif (AP2-2a_mut, open circles) the reaction did not occur.

In order to investigate the specificity of *S. agalactiae* SrtA in pilus cell wall anchoring, we tested *in vitro* the enzymatic activity of SrtA_ΔN40_ by a FRET assay using fluorogenic peptides mimicking the LPXTG motifs of the other pilus 2a subunits, the backbone protein (BP-2a), that was already shown to be cleaved by PI-2a SrtC1 [20], and the major ancillary protein (AP1-2a) (Table 1). The polypeptide substrates were incubated with SrtA_ΔN40_ in the presence of NH_2_-Gly_3_, and monitored over time at 490 nm. A fluorescent signal was observed exclusively in the presence of AP2-2a peptide (Figure 5). By contrast, no increase in fluorescence was revealed in the presence of BP-2a and AP1-2a peptides, with a substrate concentration up to 240 µM, suggesting that SrtA_ΔN40_ can recognize and cleave the LPXTG-motif of the pilus 2a ancillary protein 2 but not of the backbone protein or the ancillary protein 1.

**Table 1:** Sequence of PI-2a fluorescent peptides.

Name	Sequence	MW
BP-2a	Dabcyl-KKVT**IPQTG**GIGT-Edans	1800
AP1-2a	Dabcyl-KGI**IPKTG**GK-Edans	1497
AP2-2a	Dabcyl-SF**LPKTG**M-Edans	1381
AP2-2a_mut	Dabcyl-SF**IPKTG**M-Edans	1381

(L/I)PXTG motif is shown in bold and underlined.

**Figure 6:**
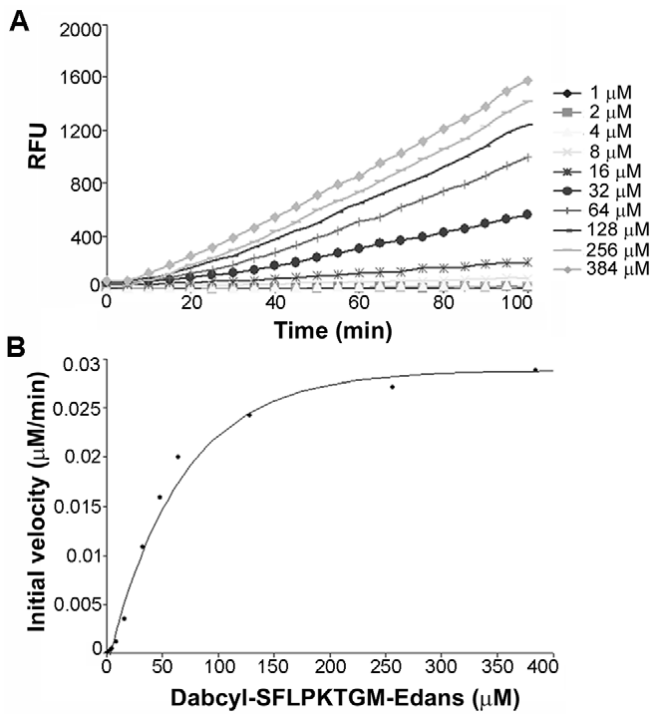
Kinetic parameters of SrtA reaction with AP2-2a peptide. (**A**) Progress curves of the transpeptidation reaction at various concentrations of AP2-2a peptide. The reaction solutions contained various concentrations of the peptide Dabcyl-SF**LPKTG**M-Edans (1, 2, 4, 8, 16, 32, 48, 64, 128, 256 and 384 µM), 75 mM triglycine and 3 µM SrtA_ΔN40_. (**B**) Steady-state rate plotted against the substrate concentration generated a Km of 48.40 µM for the transpeptidation reaction.

In order to determine the *in vitro* kinetic parameters of SrtA_ΔN40_ for the LPXTG motif of AP2-2a, we performed a kinetic analysis of the sortase catalyzed transpeptidation reaction. Figure 6A shows a set of progress curves at various concentrations of AP2-2a peptide when the triglycine concentration was fixed at 75 mM. For each progress curve in figure 6A, the converted substrate was less than 10%, representing the initial rate of the reaction. Plotting the rate vs d-SFLPKTGM-e concentration to the Michaelis-Menten equation generated an apparent K_m,app_ of 48.40 µM for the transpeptidation reaction (Figure 6B). Since the *K*_m,app_ for the peptide was determined in the presence of saturating triglycine, it can be considered as *K*_mPep_, the Michaelis constant specific for the peptide.

These results suggest that housekeeping SrtA shows a substrate-specificity only to the sorting signal of the minor ancillary protein. By contrast, the enzyme is not able to catalyze the transpeptidation reaction with substrate-peptides mimicking sorting signals of the other pilus 2a subunits (the backbone protein and the major ancillary protein).

### *In vitro* SrtA_ΔN40_ activity on GBS pilus 2a proteins

To confirm the specificity of the activity of SrtA_ΔN40_ against AP2-2a, we performed the transpeptidation reaction in the same conditions described so far, but using recombinant proteins instead of synthetic peptides. The three structural proteins of pilus 2a (BP-2a, TIGR annotation SAL_1486; AP1-2a, SAL_1487 and AP2-2a, SAL_1482) were cloned without the leader sequence and the C-terminal transmembrane domain and were successfully expressed and purified as recombinant His-tagged fusion proteins.

**Figure 7:**
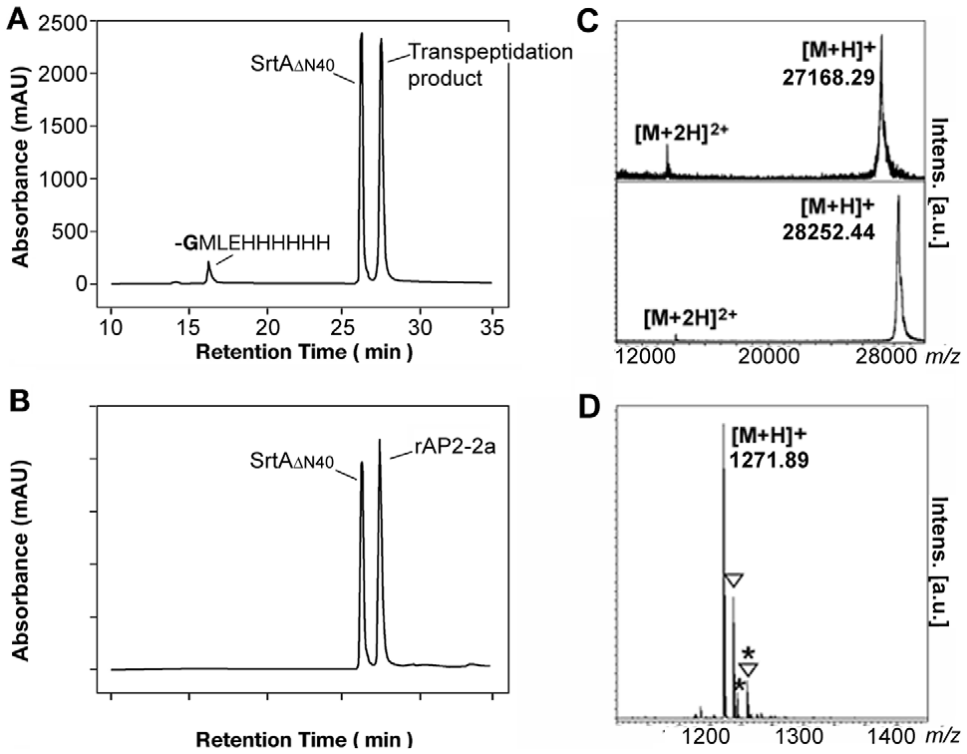
RP-HPLC profile and MALDI-TOF mass spectra of sortase reaction with recombinant AP2-2a protein. (**A**) Overnight incubation at room temperature of the reaction solution. (**B**) Starting point time of the reaction containing 30 µM of rAP2-2a, 30 µM of SrtA_ΔN40_ and 5 mM of triglycine. Chromatographic tracing corresponds to UV absorption at 215 nm of a 100 µl aliquot assay. (**C–D**) Mass spectral analysis confirmed the presence of transpeptidation products after an overnight incubation at room temperature. The reaction solution contained 30 µM of rAP2-2a, 30 µM of SrtA_ΔN40_ and 5 mM of triglycine. C upper panel shows the mass spectrum of the reaction product eluted at 27.84 min (shown in panel A), obtained by MALDI-TOF MS run in linear mode. In comparison, the uncleaved rAP2-2a eluted at 27.73 min (panel B) presented an *m/z* ratio of 28252.44 (C lower panel), in agreement with the expected average molecular mass of the recombinant protein with the lack of the initial methionine (28251.79 Da). Panel D shows the *m/z* signal of 1271.89 obtained in refectron mode from the reaction product eluted at 16.54 min (panel A), consistent with the theoretical monoisotopic mass of the peptide GMLEHHHHHH (1270.55 Da), generated by the rAP2-2a C-terminal cleavage after the transpeptidation reaction. Asterisks (*), oxidized form of the molecule (+16 Da). Triangles (**∇**), sodium adduct (+22 Da).

When SrtA_ΔN40_ was incubated with rAP2-2a in the presence of the peptidoglycan analogue triglycine, significant transpeptidation was observed (Figure 7A–B). A reaction peak collected at 27.84 min (Figure 7A) revealed an *m/z* signal of 27168.29 (Figure 7C upper panel) consistent with the theoretical mass of the transpeptidation product of rAP2-2a, in which the threonine residue of the LPXTG motif was cleaved and the triglycine added (expected average molecular mass 27169.58 Da, with the lack of the initial methionine). In comparison, the uncleaved rAP2-2a was eluted at 27.73 min (Figure 7B) and an *m/z* ratio of 28252.44 was observed (Figure 7C lower panel), consistent with the expected average molecular mass of 28251.79 Da (lacking the initial methionine). The other reaction peak eluted at 16.54 min (Figure 7A) and revealed an *m/z* signal of 1271.89 (Figure 7D), consistent with the theoretical monoisotopic mass of the peptide GMLEHHHHHH (1270.55 Da), generated by the rAP2-2a C-terminal cleavage during the transpeptidation reaction.

Neither full-length rAP2-2a substrate nor products from SrtA_ΔN40_ catalyzed LPXTG hydrolysis were detected, showing that the reaction proceeded completely and exclusively towards transpeptidation products. A comparison of the area of the peak at 16.54 min with the standard curve constructed using various concentrations of a synthetic peptide GMLEHHHHHH, confirmed that all of the substrate which was added to the reaction had been converted (data not shown).

**Figure 8:**
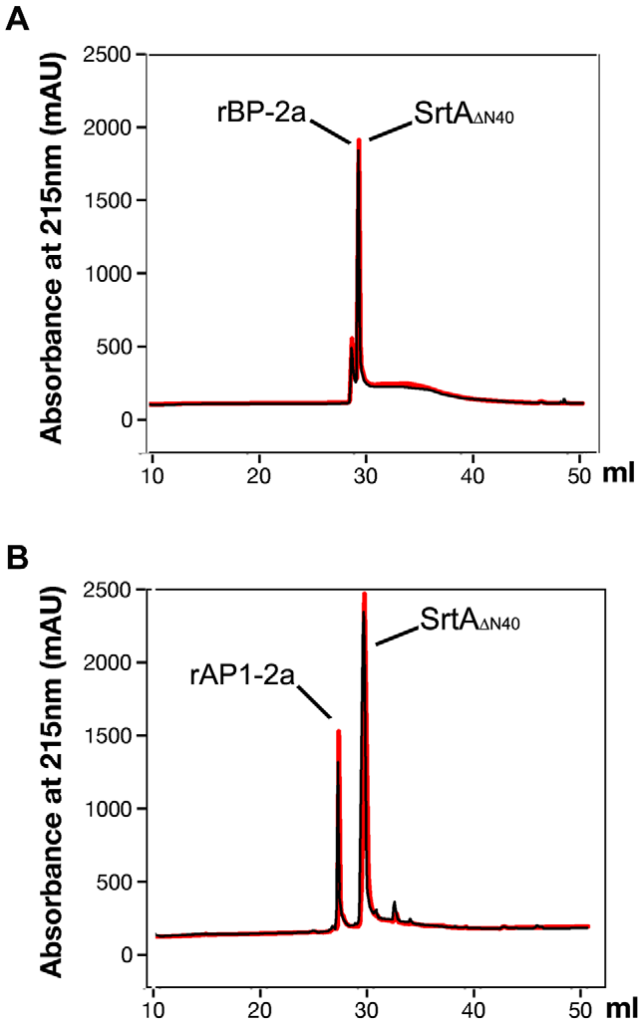
Sortase reactions with rBP-2a and rAP1-2a. Reactions containing 30 µM of SrtA, 5 mM of triglycine and 30 µM of rBP-2a (panel A) or 30 µM of rAP1-2a (panel B) were analysed by RP-HPLC. Comparing time zero (red trace) and the overnight incubation at RT (black trace) of these reactions, we did not observe an extra peak due to the cleavage of PI-2a protein subunits by SrtA.

When the transpeptidation reaction was repeated in the same conditions using rBP-2a or rAP1-2a as substrates in place of rAP2-2a, no new peaks were observed by RP-HPLC analysis after overnight (o/n) incubation (Figure 8), demonstrating that the transpeptidation can occur only with the rAP2-2a protein. Furthermore, at the end of the reaction with rBP-2a, the collected peaks were analysed by MALDI-TOF MS showing that they contained only the original unmodified substrate protein. MALDI-TOF MS analysis was also performed for the peaks collected from the sortase reaction with rAP1-2a. Although rAP1-2a was not ionized, there was no peptide resulting from the sortase cleavage in the reaction mixture after o/n incubation (data not shown).

## Discussion

In this study we provide the first biochemical characterization of GBS housekeeping SrtA related to its role in pilus cell wall anchoring. In Gram-positive bacteria pilus assembly occurs by a two-step mechanism, where pilin subunits are polymerized in high molecular weight (HMW) complexes by class C pilin-specific sortases and then covalently anchored to the cell wall peptidoglycan. As reported for *C. diphtheriae* [18], [22], [31] and GBS itself [12], [17] cell wall anchoring of pili is mediated by the housekeeping sortase and the minor ancillary pilin, acting as the pilus anchor. In this paper, by *in vivo* mutagenesis studies we demonstrated that the minor ancillary protein of pilus type 2a (AP2-2a) anchors the pilus to the bacterial cell wall through its LPXTG sorting signal. This is in accordance with data already reported for *C. diphteriae* [31]. To better investigate the SrtA specificity in the pilus cell wall anchoring step, we successfully produced the recombinant enzyme in the active form, which was able to catalyze the *in vitro* hydrolysis and transpeptidation reactions using a fluorescent quenched polypeptide carrying the LPXTG motif of AP2-2a subunit. It was interesting to observe that the GBS SrtA *in vitro* activity was independent of calcium ions. This is different to data observed for the highly characterised housekeeping SrtA in *S. aureus* [28]. Furthermore, a sequence and structural comparative analysis between *S. aureus* and GBS SrtA enzymes revealed significant differences. Specific residues identified in the region corresponding to the calcium binding cleft in the *S. aureus* enzyme structure, such as the two Glu residues (Glu105 and Glu108) and Asp112 on the β3/β4 loop and Glu171 on the β6/β7 loop [28], were not conserved in the sequence of the GBS SrtA enzyme. Moreover, the entire negatively charged calcium binding cleft of the *S. aureus* enzyme was not identified in the GBS SrtA structural model, and the structural organization of the β6/β7 loop, which in *S. aureus* SrtA_ΔN59_ is located immediately above the catalytic pocket, was noted to cause less obstruction to the catalytic domain in the GBS enzyme. These observations could be translated in different reaction kinetics of SrtA enzymes in the two species. Our structural predictions were further confirmed by NMR spectroscopy, which showed that SrtA_ΔN40_ did not bind to metal ions present in the culture broth or to calcium ions added to the protein solution.

By exploring the specific role of GBS SrtA in pilus 2a assembly we have observed that the enzyme processed exclusively the LPXTG motif of AP2-2a, the putative pilus anchor protein, but not the sorting signals of the backbone protein (BP-2a) or the major ancillary protein (AP1-2a), being conversely both proteins substrates of pilus-specific class C sortases. As a matter of fact, using a FRET assay we had previously observed that a recombinant pilus-specific sortase C1 (SrtC1) was able to hydrolyze synthetic peptides mimicking the LPXTG motif both of the BP [20] and of the major ancillary protein (AP1-2a) (unpublished data).

The analysis of the transpeptidation products by MALDI-TOF MS demonstrated that the recombinant SrtA_ΔN40_ in the presence of triglycine catalyzed specifically the transpeptidation reaction on the peptide bond between the threonine and the glycine of the LPXTG motif of AP2-2a. Neither residual substrate nor hydrolysis products were identified, suggesting that the substrate peptide was completely processed and the reaction proceeded exclusively towards transpeptidation. To understand if the activity and specificity of SrtA for the LPXTG motifs of AP2-2a, AP1-2a and BP-2a could be substrate concentration dependent, we investigated the enzymatic activities towards various concentrations of fluorogenic substrates. We measured the kinetic constants by FRET assay, using the peptides carrying the LPXTG motifs of the three pilus subunits. We operated at concentrations within linearity range and we constructed a calibration curve incorporating the inner filter effect, as already described by Huang and colleagues [27]. Our kinetic analysis generated a K_m,app_ of 48.40 µM for the transpeptidation reaction with AP2-2a peptide. By contrast, SrtA_ΔN40_ was not active on BP-2a or AP1-2a peptides for substrate concentrations at least 10-fold greater than the K_m,app_ for AP2-2a peptide.

The specificity of the transpeptidation activity of GBS SrtA_ΔN40_ was also demonstrated by performing reactions with pilus 2a recombinant full length proteins, instead of synthetic peptides, and analyzing them by RP-HPLC. The correct transpeptidation product was found and identified by MALDI-TOF MS-spectra exclusively in the reaction with the ancillary protein rAP2-2a. By contrast, no hydrolysis/transpeptidation occurred in reactions with the pilus backbone protein rBP-2a or the major ancillary protein rAP1-2a.

All of this data confirmed our hypothesis that both the backbone protein and the major ancillary protein are not substrates of the housekeeping SrtA, which encompass its role in pilus 2a cell wall anchoring by acting exclusively on the minor ancillary protein. These findings are in agreement with the current model of pilus assembly/anchoring in GBS and, in general, in Gram-positive bacteria, in which pilus-related SrtC enzymes are responsible for polymerization of pilus proteins, while the housekeeping SrtA is required for covalently anchoring the entire polymerized structure to the bacterial cell wall, acting specifically on the LPXTG sorting signal of the minor ancillary protein. Therefore, the minor subunit would be the terminal subunit located at the base of the pilus. Furthermore, our previous expression data for each pilin subunit support the proposed pilus anchoring mechanism. FACS analysis performed on whole bacteria clearly demonstrate that, among pilin subunits, only the minor ancillary proteins are not surface-exposed, hence are not accessible to specific antibodies [17]. In addition, it is known that all pilus proteins from different pilus types are promising vaccine candidates with the exception of the minor pilin subunits [32] and this evidence seems tightly in agreement with their function as anchor proteins at the base of pili. The same topological organisation seems to be shared with other Gram positive bacteria, as demonstrated for *C. diphteriae* [22] and from the recently determined *S. pneumoniae* pilus structure [23], showing the minor accessory subunit at the proximal end to the cell wall.

Given that pilus biogenesis is a highly regulated process, we speculate that the pilus polymerization stage is terminated when the minor ancillary protein is incorporated into the growing structure from the pilin-specific sortase. Subsequently, it is recognized by the housekeeping sortase A and it is irreversibly linked to the bacterial cell wall.

Although we believe that our data provide clear insights into a GBS pilus 2a anchoring mechanism, it remains to be investigated if this proposed mechanism of pilus assembly and cell wall anchoring is conserved among different pilus types in GBS. Given the importance of pili to bacterial pathogenesis and vaccine strategies, elucidating in more detail the anchoring mechanisms of PI-1 and PI-2b will remain a priority and additional studies are currently ongoing.

## Materials and Methods

### Ethics Statement

Animal treatments were performed in compliance with the Italian laws, and approved by the institutional review board (Animal Ethical Committee) of Novartis Vaccines and Diagnostics, Siena, Italy.

### Bacterial strains, plasmids and growth conditions

GBS strain 515 (serotype Ia) was used as a source of DNA for cloning genes coding SrtA and PI-2a LPXTG proteins. The GBS-knockout (KO) mutant strain for the minor ancillary protein (Δ*AP2-2a*) was generated as previously reported [17]. Bacteria were cultivated at 37°C and 5% CO_2_ in Todd Hewitt broth (THB) or in chemically defined FMC medium [33].

*Escherichia coli* DH5α (Invitrogen) was used for cloning purposes and *E. coli* BL21 (DE3) for expression of recombinant proteins. *E. coli* cells were grown aerobically at 37°C in Luria-Bertani medium (LB) in the presence of 100 µg/ml ampicillin. The expression of the ^15^N enriched protein for NMR analysis was achieved by growing cells in M9 minimal media containing 1 g/l of [^15^NH_4_]_2_SO_4_ (Sigma), 3 g/l of glucose as a unique nitrogen source, and supplemented with CaCl_2_, MgSO_4_, thiamine and biotin.

### DNA manipulation and site-directed mutagenesis

Genomic DNA was isolated from GBS strain 515 by mutanolysin-treatment of bacterial cells using a NucleoSpin Tissue kit (Macherey-Nagel), according to the manufacturer's instructions. PCR amplifications were performed using PrimeSTAR HS DNA Polymerase (Takara) and oligonucleotides listed in Table 2. Plasmids and PCR products were purified using a Wizard Plus SV Miniprep System and a Wizard SV Gel/PCR Clean-Up System (Promega).

**Table 2:** Primers used in this study.

Primer	Sequence (5′ to 3′)	Restriction site
SrtAΔN40.F	GGAATTCCATATGGCTCATCAATCAAATCATTATC	NdeI
SrtAΔN40.R	GTGGTGCTCGAGATTAATTTGATTATATTTTTTCG	XhoI
rAP2-2a.F	CTCTCTCGCTAGCGATACCCCTAATCAACTAAC	NheI
rAP2-2a.R	GTGTGAGCTCGAGCATTCCTGTTTTAGGAAGAAA	XhoI
rBP-2a.F	CTCTCTCGCTAGCGAAGAAGCAAAAACTACTGAC	NheI
rBP-2a.R	GTGTGAGCTCGAGACCACCTGTTTGTGGAATAGT	XhoI
rAP1-2a.F	CACCGAAAGTACCGTACCGGAAAATGGTGC	
rAP1-2a.R	TCATTATCGTCGTCGTCGTCGTCGTCCTTTCCCACCTGTCTT	
pAM-AP2Not.F	CCTGTCATGCGGCCGCGAAAGAGAAAGGGAAATCAAA	NotI
pAM-AP2Bgl.R	GTCGGGAGATCTGCCCTGAAGACACCTATAGC	BglII
AP2ΔLPKTG.F	AATCATTTATGATTATTGGTGGAGGACTGACA	
AP2ΔLPKTG.F	ATAATCATAAATGATTGTGGAAAAAGCGGTTG	

F corresponds to forward primer and R to reverse primer. Restriction sites are underlined.

A PCR-based site-directed mutagenesis of double-stranded DNA [34] was used to generate the AP2_ΔLPKTG_ mutant. Two PCR fragments, overlapping for 15 bp and comprising of the desired mutation, were used as a template to generate a new 1023 bp DNA fragment using the primers pAM-AP2Not.F and pAM-AP2Bgl.R. The new fragment contained the *AP2-2a* coding sequence with the deletion of the LPXTG coding region. DNA sequencing confirmed the mutation. The fragment was then digested with NotI and BglII restriction enzymes and cloned into the *E. coli*-streptococcal shuttle vector pAM401/gbs80P+T [17]. The newly generated complementation plasmid pAM-AP2_ΔLPKTG_ was used to transform GBS 515*ΔAP2-2a* by electroporation. Complementation was confirmed by detection of AP2-2a expression by immunoblotting analysis.

### Immunoblotting

*S. agalactiae* strains were maintained at 37°C and 5% CO_2_ in chemically defined FMC medium [33]. Stationary-phase cells were harvested, washed in phosphate-buffered saline (PBS) and resuspended in spheroblasting buffer (20 mM Tris-HCl pH 6.8, 10 mM MgCl_2_, 26% raffinose w/v) containing 400 U of mutanolysin. Cell suspensions were incubated at 37°C for 2 hours. Protoplasts were removed by centrifugation (12,000× g for 10 min) and the supernatants, representing the cell wall fraction, were collected.

To visualize the proteins released during growth, culture supernatants were harvested (3,000× g for 20 min), dialyzed extensively against distilled H_2_O and concentrated by lyophilization, before being subjected to sodium dodecyl sulfate-polyacrylamide gel electrophoresis (SDS-PAGE). 4 ml of supernatant equivalent and corresponding cell pellets were resolved on 3–8% NuPage Novex SDS-PAGE gels (Invitrogen) and then transferred to nitrocellulose. Membranes were probed with mouse antiserum directed against AP2-2a and BP-2a (1∶1,000 dilution), followed by a rabbit anti-mouse horseradish peroxidase-conjugated secondary antibody (Dako, Glostrup, Denmark). Bands were then visualized using an Opti-4CN substrate kit (Bio-Rad).

The equal quantity loaded in each well is verified by immunoblotting the same gel with a control antiserum that recognizes a secreted protein PcsB of 47 kDa [35]. Specific antisera were generated by immunizing CD1 mice with recombinant proteins, as reported previously [15], [17], [32].

### Cloning, expression, and purification of recombinant proteins

Genes coding for PI-2a LPXTG proteins (TIGR annotations: SAL_1486 [BP-2a], SAL_1487 [AP1–2a], and SAL_1482 [AP2–2a) were PCR amplified using chromosomal DNA from GBS strain 515 and primers listed in Table 2. *srtA* gene (TIGR annotation SAL_1016) was PCR amplified from nucleotides 121 to 741, encoding amino acid residues 41–247 (SrtA_ΔN40_). The PCR products were cloned in pET21b (Novagen) and expressed in *E. coli* BL21(DE3) cells as 6His-tagged fusion proteins. By contrast, AP1–2a was expressed in pColdI (Takara) modified for Gateway technology (Invitrogen). After sonication in the the 10 mM HEPES (pH 8.0) lysis buffer, recombinant proteins were purified by affinity chromatography in 5 ml HiTrap Chelating HP column (Amersham). The affinity column was previously charged with NiCl_2_ and equilibrated with buffer A [10 mM HEPES, 300 mM NaCl pH 8.0]. The desired protein was eluted using a linear gradient from 20 to 250 mM imidazole. Protein fractions were pooled and 5 mM DTT was added in the case of SrtA_ΔN40_ to avoid enzyme dimerization. The final pool was loaded onto a Superdex 75 26/60 gel filtration column for SrtA_ΔN40_ and AP2-2a or onto a Superdex 200 26/60 column (Amersham) for BP-2a and AP1-2a. Gel filtration columns were pre-equilibrated with buffer B [10 mM HEPES, 50 mM NaCl, pH 7.5] or with buffer C [10 mM HEPES, 50 mM NaCl, 5 mM DTT, pH 7.5] to purify SrtA_ΔN40_.

Protein purity was verified by SDS-PAGE analysis with the Bio-Rad electrophoresis system (Bio-Rad Laboratories) using precast 12% polyacrylamide gels. Protein concentrations were determined using the Bradford method and bovine serum albumin (BSA) as a standard. The SrtA_ΔN40_ concentration was determined using the calculated extinction coefficient (A_280_) 11920 M^−1^ cm^−1^.

### SEC-MALS analysis of SrtA_Δn40_

An AKTA Purifier (GE Helthcare) chromatographic system was used for Size Exclusion Chromatography with UV detection analysis. A Superdex 200 PC 3.2/30 (GE Healthcare) column with a MW 10.000–500.000 Da separation range on globular proteins was used. Samples were eluted isocratically in PBS at a flow rate of 0.1 ml/min. UV absorbance was monitored at both 214 and 280 nm. MALS analyses were performed connected on-line in the same chromatographic conditions with detection made in the UV at 280 nm, using a Multi Angle Light Scattering Detector DawnTREOS (Wyatt Corporation, Santa Barbara, CA). The DawnTREOS incident laser wavelength was 658 nm, and the intensity of the scattered light was measured at 3 angles simultaneously; data elaboration was performed by the Software Astra V (Wyatt). Zimm formalism was used to determine the weight-average molecular mass (MW) in Da and polydispersity index (MW/Mn) for each oligomer present in solution. Uncertainties of measurements were directly calculated by Astra software.

### NMR spectroscopy analysis

^1^H,^15^N-HSQC spectra were recorded at 25°C on a Bruker Avance III spectrometer operating at 600.13 MHz proton Larmor frequency equipped with a triple resonance TCI cryoprobe incorporating z axis gradients. Standard ^1^H-^15^N HSQC pulse sequences were used, which employ pulsed field gradients to achieve suppression of the solvent signal and spectral artefacts.

The protein, treated with the metal chelating agent EDTA, was dissolved in 30 mM phosphate buffer, 1.5 mM DTT, pH 6.5. The chelating agent EDTA was added to the protein sample in increasing amounts, to a final concentration of 3 mM (largely in excess with respect to protein concentration), to check for the presence of bound metal ions derived from the cell culture broth. Before CaCl_2_ addition, the previous sample was buffer exchanged in 50 mM Hepes pH 6.5 using a PD10 column. Fractions containing protein in the new buffer were collected and concentrated to a final volume of 600 µl. Increasing amounts of CaCl_2_ were added up to a protein∶ion molar ratio 1∶10. All of the experiments were performed with a 0.3 mM sample concentration, in the presence of 7% D_2_O for the NMR spectrometer frequency lock. The ^1^H,^15^N-HSQC spectra were acquired using a spectral width of 2432.718 Hz, and 2048 complex points in the ^15^N dimension and a spectral width of 9515.385 Hz and 256 complex points in the ^1^H dimension. Processing of all the spectra were obtained with TOPSPIN 2.1 (Bruker).

### FRET assay

The FRET (Fluorescence Resonance Energy Transfer) assay was used to monitor the *in vitro* activity of the recombinant SrtA_ΔN40_. We used fluorescently self-quenched peptides, tagged with Edans as a fluorophore and Dabcyl as a quencher, containing the sorting signals of pilus 2a subunits (Table 1). All synthetic fluorogenic peptides listed in Table 1 were purchased from Thermo Scientific Biopolymers and they were dissolved in 50% DMSO. The activity test was performed in a 200 µl reaction mixture containing 20 mM HEPES (pH 7.5), 3 µM SrtA_ΔN40_, 192 or 256 µM fluorogenic peptide and when specified 75 mM triglycine (Sigma). When reported 5 mM CaCl_2_ was added to the assay buffer. Reactions were started by the addition of the enzyme and they were monitored by measuring the increase in fluorescence every 5 minutes (λex = 336 nm, λem = 490 nm) at 37°C on an InfiniteM200 Spectrophotometer microplate reader (TECAN). Assays were carried out in 96F black microplates for fluorescence reading (NUNC-Thermo Fisher Scientific).

### Kinetic measurements

All kinetic data were obtained by incubating various concentrations of peptides with a constant enzyme concentration to achieve between 5 and 20% cleavage of the substrate in each reaction. The concentration of SrtA_ΔN40_ in each reaction was 3 µM, while peptide concentrations ranged from 1 µM to 384 µM (AP2-2a peptide) and from 1 µM to 240 µM (BP-2a and AP1-2a peptides).

All reactions were performed at 37°C in 20 mM HEPES pH 7.5 in the presence of 75 mM triglycine (Sigma). Reactions were initiated by the addition of the enzyme and they were monitored by measuring the increase in fluorescence every 5 minutes for 100 minutes (λ_ex = _336 nm, λ_em = _490 nm) on an InfiniteM200 Spectrophotometer microplate reader (TECAN).

To correlate the fluorescence signal, expressed in Relative Fluorescence Unit (RFU), with the concentration, the standard curves of the fluorophore NH_2_-GM-Edans in the absence and presence of an equal concentration of the quencher Dabcyl-SFLPKT-OH were collected. The presence of Dabcyl-SFLPKT-OH clearly decreased the fluorescence of NH_2_-GM-Edans. However the quenching effect was minimal when the quencher concentration was below 10 µM. The linear segment of the fluorophore standard curve generated a conversion ratio of 600 RFU/µM NH_2_-GM-Edans. Parameters containing RFU have been converted to micromolar using this conversion ratio.

Initial velocities (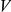) were determined from the progress curves and plotted against substrate concentration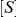. The data were fitted to the Michaelis–Menten equation 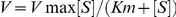 with a nonlinear regression analysis program. The best fit of the data produced 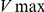 and 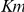 values, where 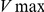 represents the maximum rate of transpeptidation and 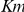 is the Michaelis constant. Assays were carried out in 96F black microplate for fluorescence reading (NUNC-Thermo Fisher Scientific).

To analyse the complete reaction products by HPLC and MALDI-TOF MS, the transpeptidation assays were carried out in 200 µl reaction mixtures containing 20 mM HEPES pH 7.5, triglycine (5 mM), SrtA_ΔN40_ (30 µM) and fluorogenic peptide (20 µM) or pilus 2a recombinant proteins (30 µM) as substrates. Reactions were started by the addition of the enzyme; mixtures were incubated overnight at 25°C.

### HPLC analysis

100 µl aliquots of the reactions were injected onto a Vydac Reverse-phase C4 HPLC column (4.6×250 mm, 5 µ particle size). Separation was achieved using a gradient of 2–80% acetonitrile in the presence of 0.1% trifluoroacetic acid (TFA) during a 36-min interval. Elution was monitored with UV detection at 280, 215 and 254 nm for the SrtA reaction with recombinant proteins. In the reaction with fluorogenic peptide, Dabcyl-containing peaks were detected by absorbance at 472 nm and Edans-containing peaks were detected by absorbance at 336 nm. To confirm the composition and identity of each product, the peaks were collected and analysed by MALDI-TOF MS.

### Mass spectrometry analysis

The determination of protein and peptide molecular masses was performed using a MALDI-TOF/TOF mass spectrometer UltraFlex (Bruker Daltonics, Bremen, GmbH). Ions generated by laser desorption at 337 nm (N_2_ laser) were recorded at an acceleration voltage of 20 kV in the linear mode for proteins and 25 kV in the reflector mode for peptides. In general, about 200 single spectra were accumulated for improving the signal/noise ratio and analysed by FlexAnalysis (version 2.4, Bruker Daltonics). 1 µl of protein solution (20–60 pmoles) was added to 1 µl of a saturated solution of sinapinic acid (3,5-dimethoxy-4-hydroxy-trans-cinnamic acid) in 30% (vol/vol) acetonitrile, 0.1% (vol/vol) trifluoroacetic acid (TFA). 1 µl of protein/matrix mixture was spotted on a stainless steel sample target and air-dried at room temperature. Protein mass spectra were calibrated using external Protein Calibration Standard II purchased from Bruker Daltonics. 0.6 µl of peptide solution (approximately 1pmole) was spotted on a matrix PAC target (Prespotted AnchorChip 96, set for Proteomics, Bruker Daltonics). Spots were washed with 0.6 µl of 70% (vol/vol) ethanol, 0.1% (vol/vol) TFA. Peptide mass spectra were externally calibrated using the standards pre-spotted on the target.

Processing of all the spectra were obtained with FlexAnalysis and Biotools (Bruker).

### Homology modeling of GBS sortase A

All molecular simulations were performed using Discovery Studio 2.5 software from *Accelrys*, USA. The aminoacid sequence of sortase A from strain 515 (SAL_1016) was used to search against the Protein Data Bank (PDB) with the BLAST program tool [36]. The best template structure found is PDB code 3FN5 (chain A) corresponding to the crystal structure of *Streptococcus pyogenes* spy_1154 (residues 69 to 249). Pairwise sequence alignment between SAL_1016 and spy_1154 was done using *multiple sequence alignment* tools in DS modeling 2.5. The model was generated with MODELLER [37] from *Protein modeling* module of DS 2.5, performing both homology modeling and loop refining for the protein. Ten models have been generated and the model which shared the least RMS deviation with respect to trace (Cα atoms) of the crystal structure of the template was selected for further refinements and validations. The quality of the refined SAL_1016 structure obtained was checked with *Verify profile-3D* module in DS2.5, and its stereochemical quality was examined by *Protein health* tools of DS2.5.
